# Colorectal Surgery in Cirrhotic Patients

**DOI:** 10.1155/2014/239293

**Published:** 2014-01-15

**Authors:** Jacqueline Paolino, Randolph M. Steinhagen

**Affiliations:** ^1^Icahn School of Medicine at Mount Sinai, New York, NY 10029, USA; ^2^Division of Colon and Rectal Surgery, Department of Surgery, Icahn School of Medicine at Mount Sinai, P.O. Box 1259, One Gustave L. Levy Place, New York, NY 10029, USA

## Abstract

Patients with cirrhosis have a greater risk of morbidity and mortality following colorectal surgery. Therefore, preoperative medical optimization and risk assessment using criteria such as the MELD score are vital in preventing complications. Some risk factors include age, urgency of surgery, and ASA score. Postoperative morbidity and mortality are related to portal hypertension, ascites, infection, and anastomotic and stomal complications. This review highlights the assessment of risk and perioperative management of cirrhotic patients undergoing colorectal surgery.

## 1. Introduction

Cirrhosis represents the end stage of damage to hepatocytes, and has many etiologies ranging from viral to toxic to autoimmune. It is the 12th leading cause of death in the United States. Patients are classified as compensated or decompensated, with a median survival of 12 years and 2 years, respectively. Signs of decompensated cirrhosis include portal hypertension, seen clinically as varices and ascites, jaundice, encephalopathy, coagulopathy, renal impairment, and infections including spontaneous bacterial peritonitis [1].

Cirrhotic patients are well known to experience a higher risk of morbidity and mortality following surgery. The severity of liver disease plays a role in the increased risk, as well as age, urgency of surgery and comorbidities. Colorectal surgery has been associated with a higher perioperative risk [2]. Complications from anastomoses and stoma creation represent unique concerns in this population, as well as risk of infection, ascites, and decompensation of liver disease. This review will highlight the assessment of risk and perioperative management of cirrhotic patients undergoing colorectal surgery.

## 2. Perioperative Management of Cirrhotic Patients

Cirrhotic patients are at a higher risk for postoperative morbidity and mortality following any operation. Many risk factors have been identified, such as older age, higher American Society of Anesthesiologists (ASA) score, emergency operation, intraoperative blood loss, preoperative ascites, hyponatremia, and hypoalbuminemia [3, 4]. The types of operation also affect the risk, with cardiac surgery and open abdominal surgeries including cholecystectomy, gastric resection, colectomy, and hepatic resection conferring the highest risk [2].

Preoperative optimization and management of comorbidities are very important for the improvement of outcomes. Correction of coagulopathy and thrombocytopenia, improvement of renal and respiratory function and nutrition status, and treatment of encephalopathy, ascites, and electrolyte imbalances are all important interventions in the preoperative period. For patients with coagulopathy, administration of fresh frozen plasma and vitamin K to a prothrombin time within 3 seconds of normal has been recommended. Platelet transfusion is recommended to increase platelets over 50,000/mm^3^. Encephalopathy has many causes and is often multifactorial; therefore, treatment should be directed at fixing the underlying causes including infection, electrolyte disturbance, constipation, medication, hypoxia, sepsis, and bleeding [5].

Perioperative fluid management is a particular challenge in cirrhotic patients undergoing surgery. Telem et al. report that blood loss over 150 mL had an odds ratio of 3.9 and intraoperative transfusion requirement had an odds ratio 16.8 for postoperative morbidity and mortality. They posit that increased blood loss might be attributable to technical difficulty of surgery, and that transfusion may be an indirect measure of the same problem, or might impede recovery due to effects on the immune system [4]. Neeff et al. also demonstrated that intraoperative transfusion was an independent predictor of increased mortality [6]. The accumulation of postoperative ascites can be prevented by restricting sodium and minimizing intravenous fluid replacement [7]. In all patients irrespective of liver function, perioperative fluid restriction leads to better outcomes, with shorter hospital stays, speedier return of bowel function, and fewer complications [8]. These data support the restriction of fluid and transfusion in cirrhotic patients to prevent postoperative morbidity.

## 3. Portal Hypertension

Portal hypertension is an important feature of decompensated cirrhosis and can be seen clinically as the development of varices and ascites. It is defined as a portal pressure above 6 mmHg, which is indirectly determined by the hepatovenous portal gradient (HVPG) across the liver. Esophageal varices are formed at HVPG over 10 mmHg, and, in patients who maintain a HVPG <10 mmHg, the negative predictive value is 90% for decompensating clinically. For every 1 mmHg increase in HVPG, there is a 3% increase in mortality, and 1 year mortality for an HVPG >20 mmHg is 64% compared to 20% for <20 mmHg. Reducing the HVPG by 10% may prevent development of varices, and a 20% reduction is associated with a 60% reduced mortality [1]. Portal hypertension places cirrhotic patients who undergo abdominal surgery at higher risk of complications postoperatively. Surgery can lead to dilation of collateral vessels, causing reflexive hypotension and end organ ischemia [9]. Venous congestion also increases the risk of intraoperative and postoperative hemorrhage, and ascites can lead to peritonitis or wound dehiscence. Preoperative optimization of these patients includes the use of banding to prevent variceal bleeding, fluid restriction, diuretics or large volume paracentesis to reduce ascites, and antibiotics to prevent spontaneous bacterial peritonitis. Additionally, portal decompression has been suggested to decrease perioperative morbidity and mortality [10].

Transjugular intrahepatic portosystemic shunt (TIPS) is a minimally invasive procedure used to treat the complications of portal hypertension such as refractory ascites and variceal bleeding. It has been proposed by Azoulay et al. as a possible neoadjuvant treatment for patients with portal hypertension requiring abdominal surgery. In their study, seven cirrhotic patients were treated with TIPS 1–5 months prior to abdominal surgery, with a reduction in HVPG from 18 ± 5 mmHg to 9 ± 5 mmHg. Six of the seven patients had uneventful postoperative courses, and one patient died 36 days postoperatively of terminal liver failure. Based on their data, they propose performing TIPS one month before abdominal surgery. They postulate that because TIPS relieves portal hypertension, it will decrease the risk of ascites, variceal bleeding, development of venous collaterals leading to increased intraoperative bleeding, and requirement of blood transfusion, ultimately leading to better postoperative outcomes in these patients [11]. In a retrospective review by Kim et al., 19 cirrhotic patients underwent abdominal surgery and 6 patients underwent cardiothoracic surgery. TIPS had been placed on average 20 days preoperatively, and 32% and 24% of surgeries were performed emergently or urgently, respectively. Severe ascites developed postoperatively in 29% of patients although 71% had medically controlled ascites before the procedure, and encephalopathy was seen in 17%. One-year survival was 74%. The 3 patients who died during hospitalization had MELD scores of >25. The authors conclude that TIPS may improve the risk of selecting cirrhotic patients undergoing extrahepatic surgery [9]. However, Vinet et al. retrospectively reviewed 18 cirrhotic patients who underwent TIPS 72 ± 11 days before nonhepatic abdominal surgery, and found no differences in preoperative blood loss, complications, hospital stay, and month and one-year survival operatively compared to controls [12].

TIPS is a relatively safe procedure with a mortality of 1.2% related to the procedure itself but a one-year mortality of 50%, overwhelmingly due to progressive liver failure. The potential benefits of preoperative TIPS should always be weighed against the risks of TIPS placement. The risk of hepatic encephalopathy increases following TIPS due to loss of hepatic metabolism of ammonia, and new or worsening encephalopathy is seen in 5–35% of patients. Finally, rates of post-TIPS stenosis leading to recurrent variceal bleeding and ascites are high, with 1-year incidence of 30–70% [10]. Therefore, it is often seen as a bridge to transplant, and caution should be observed in recommending TIPS for patients who are not candidates for transplant who desire elective abdominal surgery. Further studies should determine optimal patient populations and timing of the procedure.

## 4. Predictive Models for Cirrhotic Patients Undergoing Surgery

Given that patients with cirrhosis are at higher risk for surgical complications, many studies have evaluated risk prediction scores. The two most commonly used metrics of liver function in cirrhosis are the Child-Turcotte-Pugh (CTP) class and model for end stage liver disease (MELD) score. The CTP class is comprised of subjective measurements of ascites and encephalopathy as well as objective values of prothrombin time, total bilirubin, and albumin (Table 1). It has historically been used as a predictor of surgical risk. In general, CTP class A cirrhotics can undergo elective surgery, class B should be optimized prior to elective surgery, and class C should avoid surgery if possible. The MELD score is a formula based on INR, bilirubin, and creatinine. It was originally designed to predict short-term mortality in patients undergoing placement of a TIPS and is now used in prioritizing transplant allocation. In general, a MELD score under 10 roughly correlates to CTP class A in terms of surgical risk, a score of 10–15 with CTP class B, and a score over 15 with CTP class C [7]. CTP classes A, B, and C confer a postoperative mortality risk of 10%, 30%, and 80%, respectively. MELD scores of 0–11, 12–25, and >26 confer a postoperative mortality risk of 5–10%, 25–54%, and 90% [4].

Recent literature has compared the utility of both scores in assessing perioperative risk of morbidity and mortality. Befeler et al. analyzed 53 patients with cirrhosis undergoing surgery and found that a MELD score >14 was superior to Child class C in that it correctly predicted poor outcome in 77% versus 23% of cases [13]. Another group analyzed 40 cirrhotic patients who required either elective or emergency surgery and found that mortality rates were significantly higher in emergency surgery patients at one and three months. They found a correlation between CTP classes and MELD score in predicting mortality, especially in the emergency surgery group [14]. In a single-center retrospective study of 64 cirrhotic patients undergoing nontransplant abdominal and thoracic surgery, outcomes were analyzed using CTP, MELD, and MELD-Na. A CTP score of ≥7.5 was associated with an 8.3-fold increased risk of 30-day morbidity, a MELD score of ≥14.5 was associated with a 5.4-fold increased risk of 3-month mortality, and a MELD-Na score ≥14.5 was associated with a 4.5-fold increased risk of 1-year mortality. CTP had the best sensitivity and the MELD-Na score showed the best specificity [3]. Northup et al. retrospectively studied the predictive ability of the MELD score for 30-day mortality after 140 nontransplant surgical procedures in cirrhotic patients. They found that it was a good predictor for all procedures, with an average score of 23.3 in the patients who died and an average score of 16.9 in those who survived beyond 30 days. MELD score was also predictive in a subgroup analysis of 67 intra-abdominal surgeries. The authors estimated that a 1% increase in mortality per MELD point under 20 and a 2% increase over 20 could be used to estimate surgical risk [15]. These studies, while not conclusive, support the use of MELD score rather than CTP class to predict mortality and stratify perioperative risk.

Additional physiologic markers may improve the predictive ability of MELD scores. A retrospective review of 120 cirrhotic patients undergoing nonhepatic abdominal surgeries from 2001 to 2011 found that an albumin level below 3.05 mg/dL and a hematocrit below 35.55% were independent predictors of 30-mortality or transplant. When added to CTP or MELD scores, albumin and hematocrit improved the sensitivity and specificity of the prediction by 6.1 and 32.1%, respectively [16]. In a study by Telem et al., adding an albumin ≤2.5 mg/dL to a MELD score ≥15 predicted significantly increased mortality in a series of 100 cirrhotic patients undergoing abdominal surgery, with a 60% mortality rate compared to 14% in patients without those criteria [4]. Another study found that hemoglobin <10 mg/dL was an independent predictor of poor outcome equivalent to a MELD score >18 [13]. While albumin is already a criteria of the CTP class, its addition to the MELD score appears to improve risk stratification for cirrhotic patients undergoing surgery.

## 5. Colorectal Surgery in Cirrhotic Patients

Colorectal surgery represents a unique challenge in cirrhotic patients. Morbidity and mortality rates at thirty days range from 21.5 to 26% and 48 to 77%, respectively [17–19]. A study of patients undergoing colorectal procedures in the Nationwide Inpatient Sample (NIS) from 2006 to 2008 identified liver disease as the comorbidity conveying the highest risk of mortality (adjusted odds ratio of 3.02). Other risk factors for poor outcome include emergent surgery, age older than 65 years, ASA class, functional status, ascites, hypoalbuminemia, encephalopathy, anemia, preoperative radiotherapy and steroid use, total colectomy, chronic renal failure, and malignant tumor [18, 20–22]. Portal hypertension also contributes to higher postoperative mortality. In a study of patients undergoing colorectal surgery in the NIS from 1998 to 2005, those with compensated cirrhosis and cirrhosis with portal hypertension had higher in-hospital mortality than noncirrhotic patients (14% and 29% versus 5%, resp.). The combined mortality of both groups was 18%. Mortality rates for emergent and urgent colorectal procedures compared with elective procedures were also significantly higher (9.2% versus 1.8%). Postoperative complications were found to be more likely in cirrhotic patients and patients with portal hypertension (adjusted odds ratio, 1.35 and 1.82, resp.) compared to patients with no cirrhosis [23].

The MELD score is a useful model for preoperative risk assessment in colorectal surgery. Ghaferi et al. used the American College of Surgeons National Surgical Quality Improvement Project (NSQIP) to study 30,927 patients with liver disease undergoing colorectal resections from 2005 to 2007. Ascites, esophageal varices, or total bilirubin greater than 2 mg/mL were used as surrogate markers of chronic liver disease, as cirrhosis is not a documented patient characteristic in the database. Patients with a MELD of greater than 15 had a higher rate of mortality, complications, and mortality following complications than patients with a MELD under 15 [17]. Another study evaluated all patients undergoing elective and emergent colorectal surgery from the NSQIP database 2005-2006. The MELD score was an independent predictor of mortality when calculated for all patients, regardless of underlying liver disease [21].

Postoperative complications include ascites, infection, bleeding, anastomotic leaks, and stoma complications. Meunier et al. examined 41 cirrhotic patients undergoing colorectal procedures and found that postoperative infection was the biggest risk for mortality, increasing it from 11% to 53%. Ascites was the only significant factor identified that contributed to morbidity. A subgroup of 28 patients underwent procedures with anastomosis and 5 experienced an anastomotic fistula postoperatively, leading to death in 3 patients. They did not find an association with preoperative ascites; however, they postulated that ascites could worsen a perianastomotic fistula into diffuse peritonitis [19]. Stoma creation in cirrhotic patients carries many risks, including peristomal leaking, infection, difficulty closing the stoma, evisceration, and peristomal variceal bleeding [19, 24]. In a study of patients with chronic liver disease at the Mayo Clinic who underwent colectomy, 31% of patients with a stoma experienced bleeding from stomal and/or esophageal varices, while bleeding from esophageal varices occurred in 15% of the nonstoma group. Patients who experienced peristomal bleeding had a higher rate of rebleeding and transfusion than patients who experienced only esophageal variceal bleeding. They found no incidence of bleeding from perianastomotic varices. The authors concluded that a distal anastomosis is superior to a terminal stoma in patients with chronic liver disease requiring colectomy [24].

Few studies have examined the risk of cirrhosis associated with colorectal surgery for cancer. A study of 39,840 Danish patients who underwent surgery for colorectal cancer found a 30-day mortality of 8.7% in patients without liver disease compared to 13.3% in patients with noncirrhotic liver disease and 24.1% in patients with cirrhosis. Mortality was greater in patients undergoing colon cancer surgery compared to rectal cancer surgery [25]. Another study of colorectal adenocarcinoma at the Mayo Clinic from 1976 to 2001 found a 30-day mortality rate of 13%. Risk factors included higher Child class, elevated bilirubin, and prolonged prothrombin time. The 1-, 3-, and 5-year survival rates were 69%, 49%, and 35%, respectively. Risks of decreased survival were a low albumin and prolonged prothrombin time. Interestingly, TNM stage of the adenocarcinoma did not contribute to prognosis. Of the study population, only 10% developed liver metastases, concordant with other studies that report low rates of metastasis in cirrhotic patients [26]. It is proposed that the poor growth environment of cirrhotic livers is the reason for the lower rate of metastasis; however, this has not been proven.

## 6. Conclusion

In summary, cirrhosis confers a higher perioperative morbidity and mortality for colorectal surgery. Perioperative risk assessment can be performed using models such as the MELD score and physiologic markers such as albumin and hematocrit. Patients should be medically optimized before surgery to prevent complications and deserve close monitoring postoperatively for signs of decompensation. There are limited data available on specific management and risk in colorectal surgery. Studies are generally limited due to institutional volume, and the majority of the data available comes from retrospective studies of large databases using surrogate markers for liver disease rather than a true diagnosis. Furthermore, there are no large studies that examine how different etiologies of cirrhosis impact colorectal surgery. Future research should clarify the risk associated with specific operations and clinical parameters to further guide surgical management and improve outcomes for cirrhotic patients undergoing colorectal surgery.

## Figures and Tables

**Table 1:** Child-Turcotte-Pugh classification of severity of cirrhosis.

Parameter	Points assigned		
	1	2	3
Ascites	Absent	Slight	Moderate
Bilirubin	<2 mg/dL	2-3 mg/dL	>3 mg/dL
Albumin	>3.5 g/dL	2.8–3.5 g/dL	<2.8 g/dL
Prothrombin time			
Seconds over control	<4	4–6	>4
INR	<1.7	1.7 to 2.3	>2.3
Encephalopathy	None	Grade 1-2	Grade 3-4

CTP class A = 5-6 points, B = 7–9 points, and C = 10–15 points.
